# Riboflavin Primes Pepper Resistance to *Ralstonia solanacearum* Through Coordinated Antioxidant Responses and Lignin Deposition

**DOI:** 10.3390/ijms27209007

**Published:** 2026-10-11

**Authors:** Fuqiang He, Yangyang Geng, Haili Li, Xiaomei Jia, Pinxue Wang, Zixuan Guo, Xiaohan Guo, Xiangyu Deng, Zhigang Li

**Affiliations:** 1 Department of Biochemistry, Baoding University, Baoding 071000, China; 2 College of Horticulture and Forestry, Tarim University/The National and Local Joint Engineering Laboratory of High Efficiency and Superior-Quality Cultivation and Fruit Deep Processing Technology of Characteristic Fruit Trees in Southern Xinjiang, Alar 843300, China; 3 Department of Artificial Intelligence, Baoding University, Baoding 071000, China

**Keywords:** antioxidant enzymes, transcriptome analysis, lignin deposition, pepper, riboflavin, *R. solanacearum*

## Abstract

Reactive oxygen species (ROS) are pivotal regulators of plant–pathogen interactions. Comparative transcriptomic analysis of a bacterial wilt-susceptible pepper cultivar (HS03) and a resistant cultivar (HR01) revealed impaired riboflavin biosynthesis in HS03. Exogenous riboflavin application compensated for this deficiency and conferred significant protection against *Ralstonia solanacearum*. Physiological and transcriptomic analyses further demonstrated that riboflavin functions as both an inducer of ROS signaling and a modulator of defense-associated metabolism. Riboflavin treatment elicited a biphasic defense response characterized by an early H_2_O_2_ burst accompanied by dynamic changes in antioxidant enzyme activities. In particular, the activities of superoxide dismutase (SOD), catalase (CAT), and peroxidase (POD) increased markedly at 6 h after riboflavin application compared with control. Following *R. solanacearum* inoculation, riboflavin-pretreated plants exhibited higher POD, ascorbate peroxidase (APX), and CAT activities at 6 h post-inoculation compared with control. This early antioxidant response induced by riboflavin was followed by increased lignin accumulation and the transcriptional activation of genes associated with cutin, suberin, and wax biosynthesis, galactose metabolism, and tyrosine metabolism at 12 h post-inoculation. Riboflavin pretreatment also enhanced the expression of genes encoding nonexpressor of pathogenesis-related genes 1 (*NPR1*) and calmodulin (*CALM*), implicating hormone signaling and calcium-mediated pathogen recognition in riboflavin-induced resistance. Collectively, these results suggest that riboflavin primes pepper defense against *R. solanacearum* by coordinating early ROS-associated antioxidant responses and subsequent cell-wall reinforcement. Riboflavin may therefore represent a promising environmentally compatible agent for the sustainable management of bacterial wilt in pepper.

## 1. Introduction

Riboflavin is a vitamin produced by plants that acts as a coenzyme in many physiological processes [1]. Three rate-limiting enzymes including GTP cyclohydrolase II/3,4-dihydroxy-2-butanone 4-phosphate synthase (DHBPs), 6,7-dimethyl-8-ribityllumazine synthase and riboflavin synthase catalyze the core steps in the de novo biosynthesis pathway of riboflavin, and are therefore functionally indispensable for the endogenous production of this metabolite [2]. Reports showed riboflavin plays a key role in plant abiotic stress. Transcriptomic scrutiny uncovered significant enrichment in pathways like riboflavin metabolism which triggers a certain “immune effect” that helps enhance resilience to silver nanoparticle challenges [3]. Exogenously applied riboflavin enhanced oxidative defense and osmoprotectant accumulation under drought stress in maize [4]; exogenous riboflavin pretreatment ameliorates salinity stress in rice by improving non-enzymatic antioxidant systems, and maintains a favorable Na^+^/K^+^ balance [5]. Research also showed riboflavin was significantly positively correlated with nicotinamide, pyridoxine, and water-soluble protein in the face of drought stress in broomcorn millets during the flowering stage [6]. Riboflavin also participated in plant resistance to biotic stress. For example, SDS-PAGE analysis revealed exogenous riboflavin application induces rice defense against *Xanthomonas oryzae* pv. *oryzae* through related proteins compared to untreated control [7]. Riboflavin induces bean resistance against *Botrytis cinerea* by promoting hydrogen peroxide accumulation and stimulation of lipoxygenase activity [8]. Random forest algorithm analysis of untargeted metabolomics on the phloem also showed riboflavin to be linked to the whitefly resistance phenotype in wild tomato [9].

However, the resistant mechanisms mediated by riboflavin are complex. Reports have demonstrated that riboflavin-derived cofactors participate in electron-transfer reactions by promoting the assembly and activity of NADH dehydrogenase and cytochrome C oxidase, thereby enhancing mitochondrial electron transport efficiency [10,11], affecting the production of reactive oxygen intermediates (ROIs), and leading to systemic resistance and production of ROS, such as hydrogen peroxide (H_2_O_2_), superoxide, and the hydroxyl radical [12,13]. Following elicitor perception and ROS accumulation, the activation of signal transduction pathways generally leads to phytoalexin biosynthesis, reinforcement of plant cell walls, and accumulation of pathogenesis-related proteins [14].

ROS function as critical, fast-acting orchestrators that link biotic responses to plant homeostasis and development [15]. High accumulation of ROS restricts the growth of pathogenic organisms by causing localized cell death around the site of infection [16,17,18], while continuous high level of ROS is also necessary for the virulence of pathogenic fungi and can also be utilized by effectors of bacterium to facilitate infection-induced cell death [19,20]. Thus, maintaining the homeostasis of ROS in plant is important under biotic stress.

As one of the most economically important vegetable crops, pepper is severely threatened by bacterial wilt caused by *R. solanacearum* [21,22]. Chemical application remains the dominant strategy for protecting pepper plants against *R. solanacearum*, which triggers much residue in pepper fruit and soil [23,24], while chemical residue also induces irreparable damage to humans. In recent years, the use of vitamins and new agents with low toxicity to strengthen the endogenous defenses of plants constitute an attractive alternative to current chemical methods [25,26]. In the previously mentioned studies, riboflavin may have functioned either as a resistance elicitor or as a mediator of resistance signal transduction. However, only a limited number of defense markers and signaling pathways, such as the phenylpropanoid pathway, have been investigated [27,28,29,30,31]. In particular, the specific role of riboflavin in pepper resistance against *R. solanacearum* remains largely unclear [32,33,34,35].

In this study, we have undertaken a leaf injection strategy to investigate the stimulation of resistance against *R. solanacearum* in pepper after application of riboflavin. Here, we show that riboflavin positively regulates pepper resistance to *R. solanacearum* by coordinated antioxidant responses, lignin deposition and expression of defense-associated genes like NPR1. These regulatory effects were comprehensively validated through parallel assays on different resistant cultivars, transcriptome analysis, biochemical assays and quantification of secondary metabolites of lignin. These findings provide fresh insights into the role of riboflavin in the protection of pepper against *R. solanacearum*, and highlight the role of antioxidant enzymes and lignin in the riboflavin-primed pepper resistance to *R. solanacearum*.

## 2. Results

### 2.1. Riboflavin Synthesis Is Closely Related to the Pepper’s Resistance to R. solanacearum

**Figure 1:**
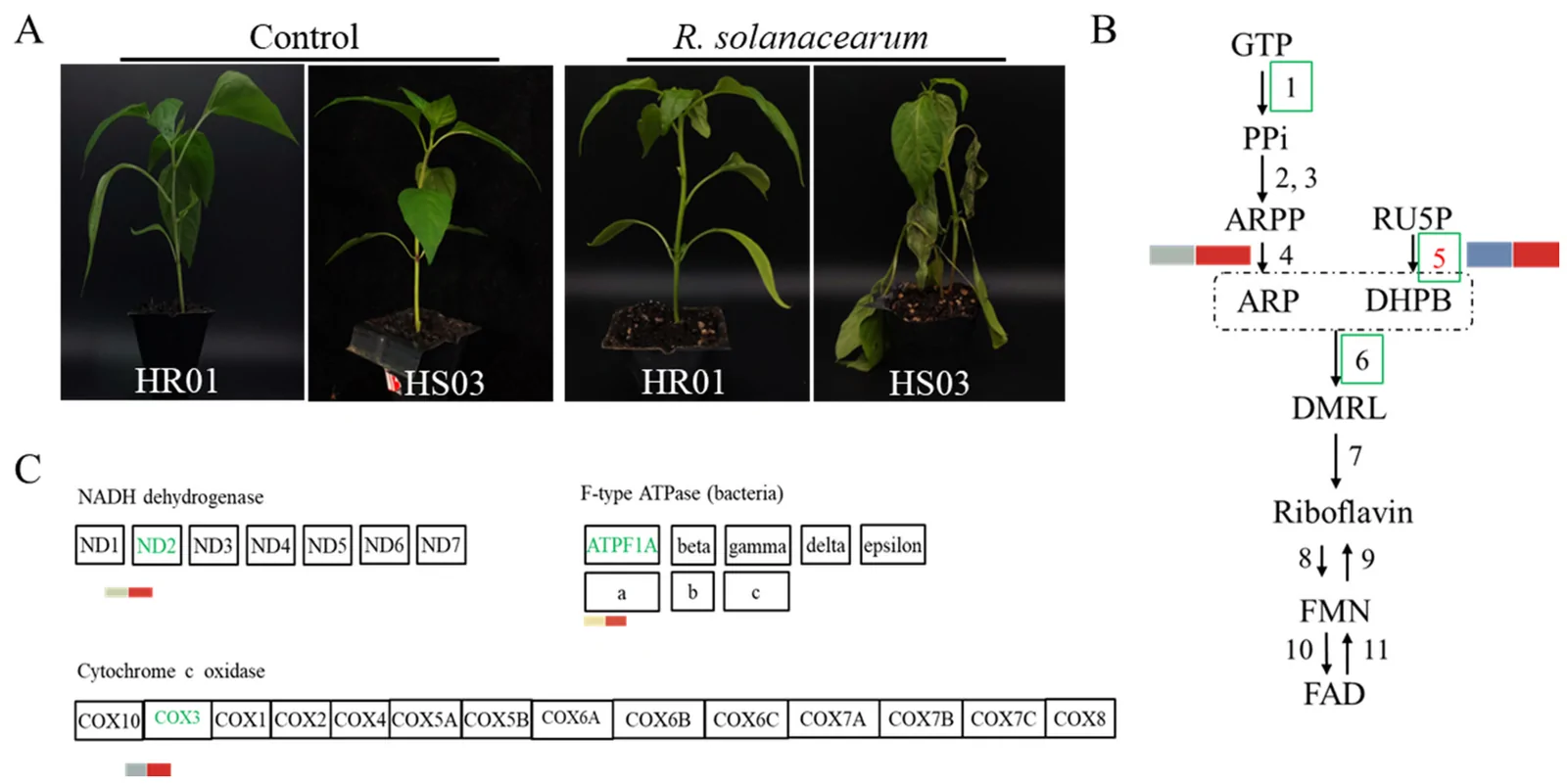
Key genes in oxidative phosphorylation pathway and riboflavin synthesis pathway were upregulated (green color) in the leaf of HR01 (resistant cultivar) compared to HS03 (susceptible cultivar) 0 hpi (hours post-inoculation) or 6 hpi. (**A**) Phenotype of HR01 and HS03 at 7 dpi with *R. solanacearum* or control at six-leave developmental stages. (**B**) Gene related to PYRP and DHBPs was upregulated in HR01 compared to HS03 at 6 hpi with *R. solanacearum*. The labels on the left of items 4 and 5 correspond to the gene expression levels of HS03; the labels on the right of these items correspond to the gene expression levels of HR01; 1 represents GTP cyclohydrolase II (GCHII, rate-limiting enzyme); 2 represents 2,5-diamino-6-ribosylamino-4-(3H) pyrimidinone-5′-phosphate deaminase, (PYRD); 3 represents 5-amino-6-ribosylamino-2,4-(1H,3H) pyrimidinedione-5′-phosphate reductase (PYRR); 4 represents 5-amino-6-ribitylamino-2,4-(1H,3H) pyrimidinedione-5′-phosphate phosphatase (PYRP); 5 represents DHBPs; 6 represents lumazine synthase (LS, rate-limiting enzyme); 7 represents riboflavin synthase (RS); 8 represents riboflavin kinase; 9 represents FMN (Flavin Mononucleotide) hydrolase; 10 represents FAD (Flavin Adenine Dinucleotide) synthase; and 11 represents FAD pyrophosphatase. (**C**) Upregulated genes of oxidative phosphorylation pathway in HR01 compared to HS03 at 6 hpi with *R. solanacearum*. The left labels represent the gene expression of HS03, and the right labels represent that of HR01. ND: NADH dehydrogenase, COX: cytochrome C oxidase, ATPF1A: ATP synthase mitochondrial F1 complex assembly factor 1.

To investigate the genes responsible for resistance to *R. solanacearum*, we collected the resistant cultivar HR01 and susceptible cultivar HS03 at the six-leaf stage. HS03 seedlings showed wilt seven days earlier than those of HR01 at the same developmental stage (Figure 1A), indicating significant resistance of HR01 against *R. solanacearum*. On the basis of the transcriptome data, key genes involved in riboflavin biosynthesis were upregulated in the leaf of HR01 compared to HS03 at 6 hpi with *R. solanacearum*, of which DHBPs represent the rate-limiting enzyme in riboflavin synthesis (Figure 1B). Furthermore, the upregulated genes in the oxidative phosphorylation pathway which tightly related to riboflavin were also observed (Figure 1C and Figure S1); additionally, the qRT-PCR revealed similar results (Figures S2 and S3). Riboflavin was subsequently selected as a candidate factor for functional analyses.

### 2.2. Riboflavin Induced Pepper Resistance to R. solanacearum at Different Concentrations

**Figure 2:**
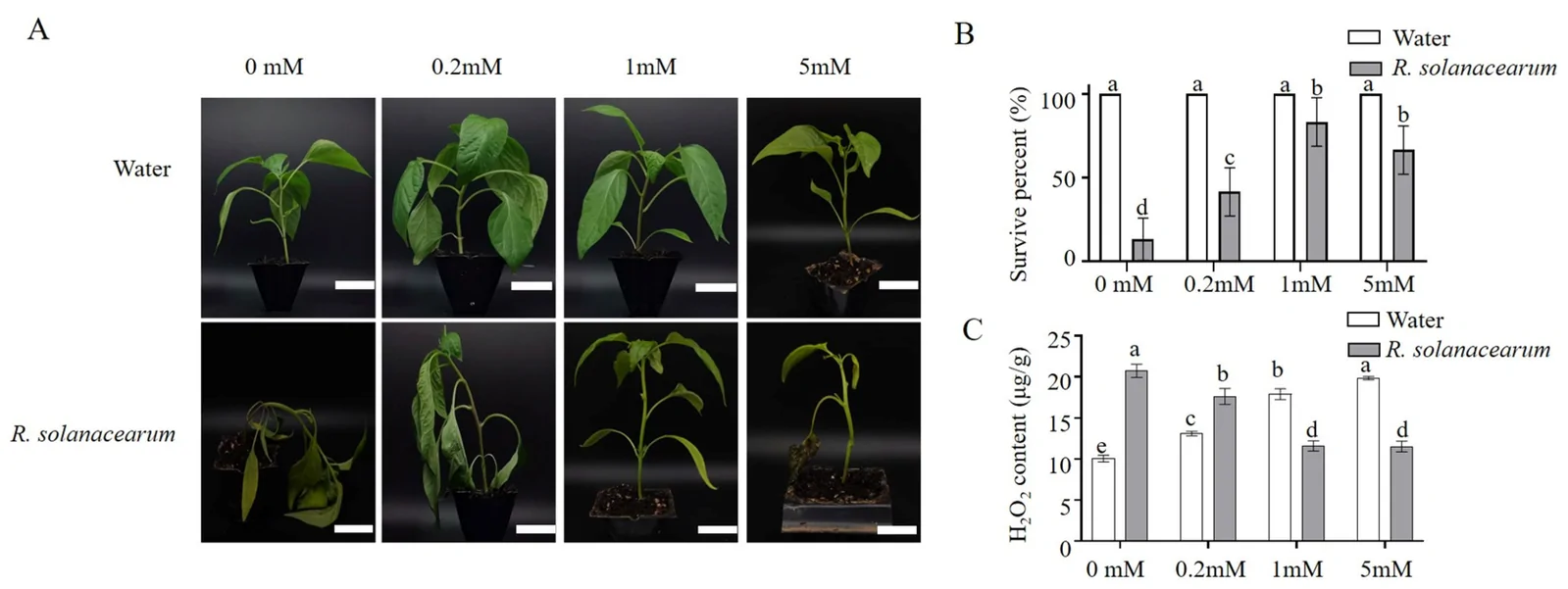
Exogenous riboflavin significantly enhanced pepper resistance to *R. solanacearum***.** (**A**) Phenotype of HS03 7 dpi with riboflavin and control (water). (**B**) Survival rate of pepper seedlings with different concentrations of riboflavin 10 dpi with *R. solanacearum*. Bars represent the standard deviations of the means (n = 3). (**C**) H_2_O_2_ content in the riboflavin-pretreated pepper leaf 6 hpi with *R. solanacearum* or water. Statistical analyses were performed using one-way ANOVA followed by Tukey’s post hoc test for normally distributed data; different letters indicate a significant difference (*p* < 0.05).

To systematically characterize the resistance-mediating effect of riboflavin against *R. solanacearum* in pepper, we conducted a concentration gradient treatment assay on the pepper cultivar HS03 using riboflavin at four distinct concentrations. Results showed that treatment with 1 mM riboflavin significantly enhanced pepper resistance to *R. solanacearum* by approximately 68% at 7 days post-incubation (dpi). In contrast, treatment with 0.2 mM riboflavin only increased resistance by approximately 7% (Figure 2A), which was consistent with the survival rate recorded at 10 dpi (Figure 2B). Notably, seedlings sprayed with 5 mM riboflavin showed earlier wilt symptoms, and no significant difference in final survival rate was observed between 5 mM and 1 mM riboflavin groups (Table S2). Results also confirmed that excessive H_2_O_2_ content accumulated in the 5 mM riboflavin pretreatment group compared with the 1 mM riboflavin treatment under water pretreatment (Figure 2A). Taken together, 1 mM riboflavin was selected for subsequent exploration. H_2_O_2_ content was subsequently verified, and the results revealed that ROS accumulated abundantly in the leaves of HS03 seedlings after 1 mM riboflavin pretreatment, followed by a well-defined H_2_O_2_ burst at 6 hpi with *R. solanacearum*. However, significantly weaker H_2_O_2_ accumulation was observed in the riboflavin-pretreated pepper seedlings than the untreated control group during the late infection stage (Figure 2C), indicating that this riboflavin actively modulates ROS homeostasis to enhance pepper resistance against *R. solanacearum*.

### 2.3. Antioxidant Enzyme Activities in Leaves

**Figure 3:**
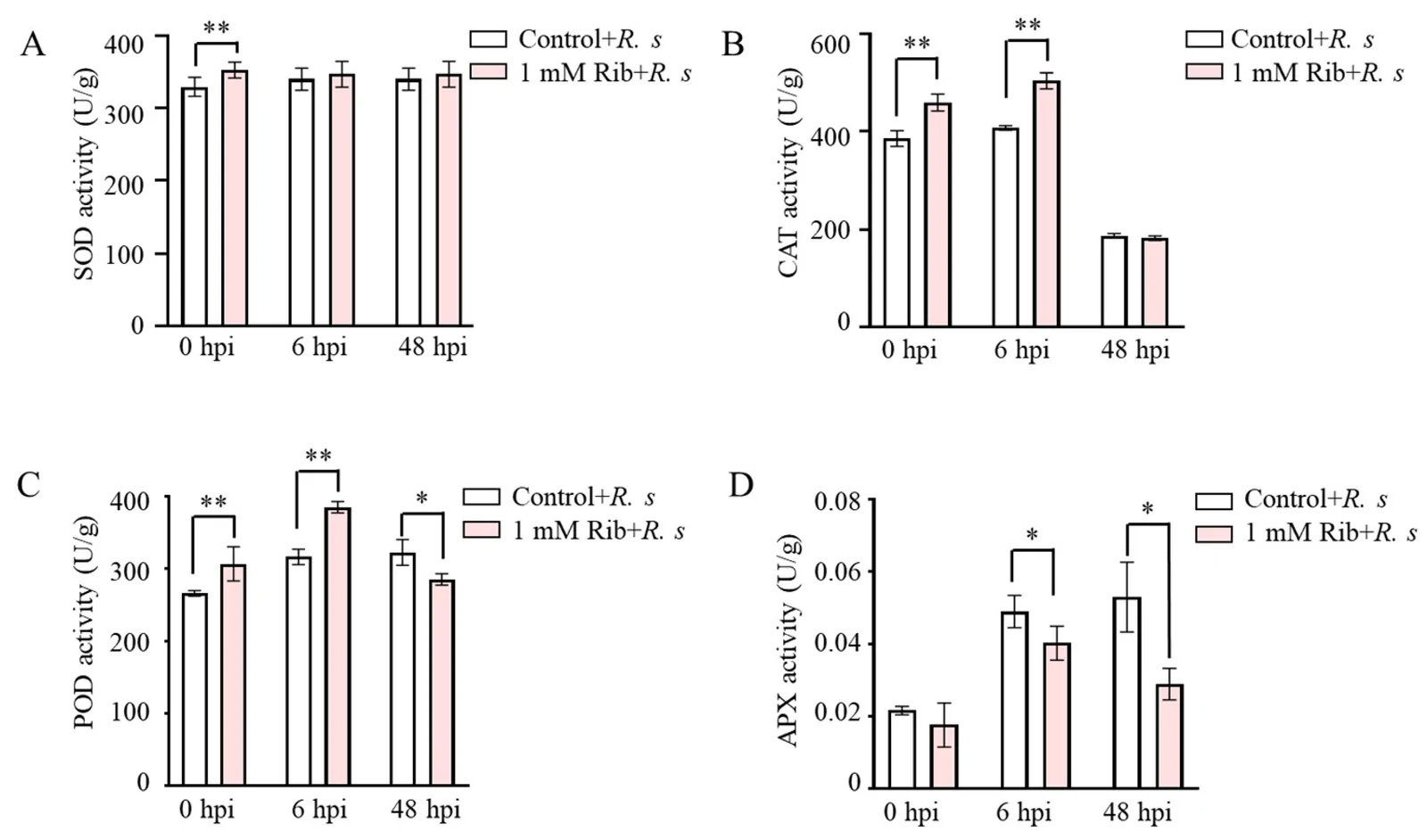
Exogenous riboflavin significantly enhanced activities of CAT and POD. Activities of SOD (**A**), CAT (**B**), POD (**C**) and APX (**D**) in functional leaves after treatment with *R. solanacearum*. The asterisks indicate statistically significant differences as determined by Student’s *t*-test (two-tailed): * *p* < 0.05, ** *p* < 0.01. Data represent mean ± SE from three independent experiments.

Riboflavin pretreatment altered antioxidant enzyme activities in pepper leaves, with responses varying among enzymes and sampling times following *R. solanacearum* inoculation (Figure 3). At 0 hpi, leaves pretreated with 1 mM riboflavin exhibited significantly higher SOD, CAT, and POD activities than the inoculated control (Figure 3A–C), whereas APX activity did not differ significantly between treatments (Figure 3D). At 6 hpi, CAT and POD activities remained significantly higher in riboflavin-pretreated leaves. In contrast, APX activity was significantly lower in these leaves, while SOD activity showed no significant difference between treatments (Figure 3A).

At 48 hpi, neither SOD nor CAT activity differed significantly between treatments (Figure 3A,B). CAT activity was lower at this time point than at 6 hpi in both groups. The difference in POD activity between treatments reversed: riboflavin-pretreated leaves exhibited significantly lower activity than the inoculated control (Figure 3C). APX activity also remained significantly lower at 48 hpi compared with control (Figure 3D). The higher antioxidant enzyme activities of CAT and POD which were induced by riboflavin at the initial stage may explain the weak ROS accumulation.

### 2.4. Transcriptome Analysis Revealed Riboflavin Induces Coordinated Gene Upregulation of Disease Resistance Signaling

**Figure 4:**
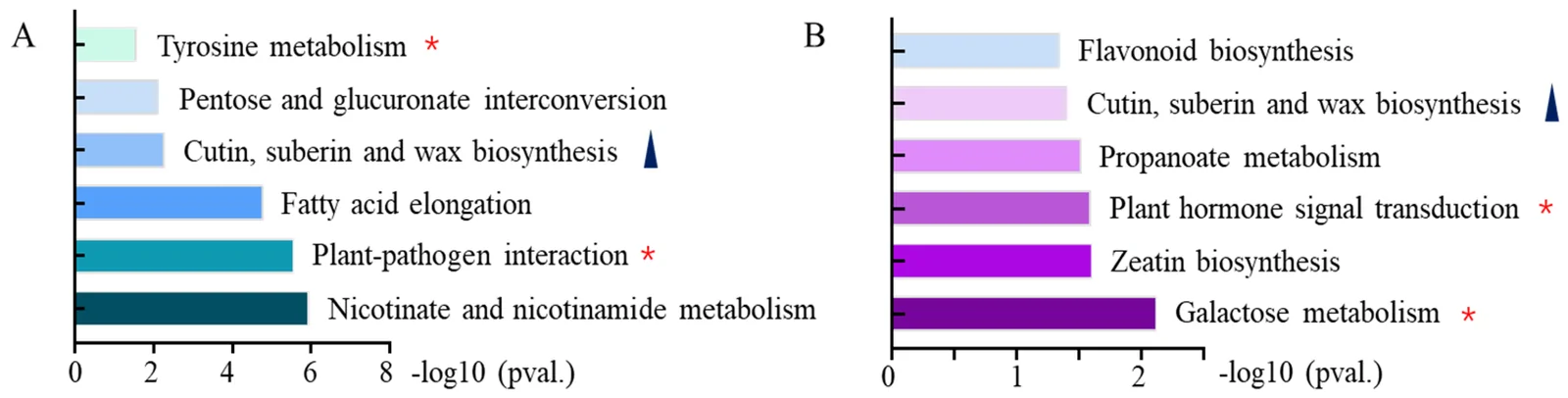
KEGG pathway enrichment analysis of genes upregulated in response to riboflavin treatment. (**A**) The major KEGG pathways enriched in riboflavin compared to control. (**B**) The major KEGG pathways enriched in riboflavin compared to control under *R. solanacearum* treatment. Red asterisks and blue triangles indicate the pathway categories highlighted in the corresponding analysis.

Qubit 3.0 package was used for quality control. A total of 42831630 clean reads were screened out for analysis. Subsequently, principal component analysis (PCA) was applied to identify significant principal components (PCs). As shown in the Venn diagrams, transcriptome analysis identified in total 432 differentially expressed genes (DEGs) in riboflavin pretreatment compared to those pretreatments with water under *R. solanacearum* treatment (Figure S4). KEGG enrichment analysis of the upregulated DEGs demonstrated that riboflavin treatment activated a broad transcriptional program associated with plant defense (Figure 4). After riboflavin treatment, the upregulated DEGs were predominantly enriched in nicotinate and nicotinamide metabolism, plant–pathogen interaction, and fatty acid elongation, with plant–pathogen interaction representing the most direct defense-related pathway. Significant enrichments were also detected in cutin, suberin, and wax biosynthesis, pentose and glucuronate interconversions, and tyrosine metabolism (Figure 4A). These pathways collectively suggest that riboflavin enhances not only pathogen-responsive signaling but also metabolic processes involved in cell-wall remodeling, protective surface-barrier formation, and defense-related secondary metabolism.

The enriched pathways in riboflavin-pretreated pepper compared with the control at 12 hpi included galactose metabolism; zeatin biosynthesis; plant hormone signal transduction; propanoate metabolism; cutin, suberin, and wax biosynthesis; and flavonoid biosynthesis (Figure 4B). Among these pathways, galactose metabolism showed the strongest enrichment, whereas the activation of plant hormone signal transduction and zeatin biosynthesis indicated extensive reprogramming of hormone-mediated defense regulation. The enrichment of flavonoid biosynthesis further suggested an enhanced capacity for antioxidant defense and the production of antimicrobial secondary metabolites. Notably, the cutin, suberin, and wax biosynthesis pathway was consistently enriched in both groups, indicating that reinforcement of the plant surface barrier may represent a conserved component of the riboflavin-induced response.

### 2.5. Riboflavin Induces Coordinated Gene Upregulation of NPR1, CALM and Lignin Accumulation

**Figure 5:**
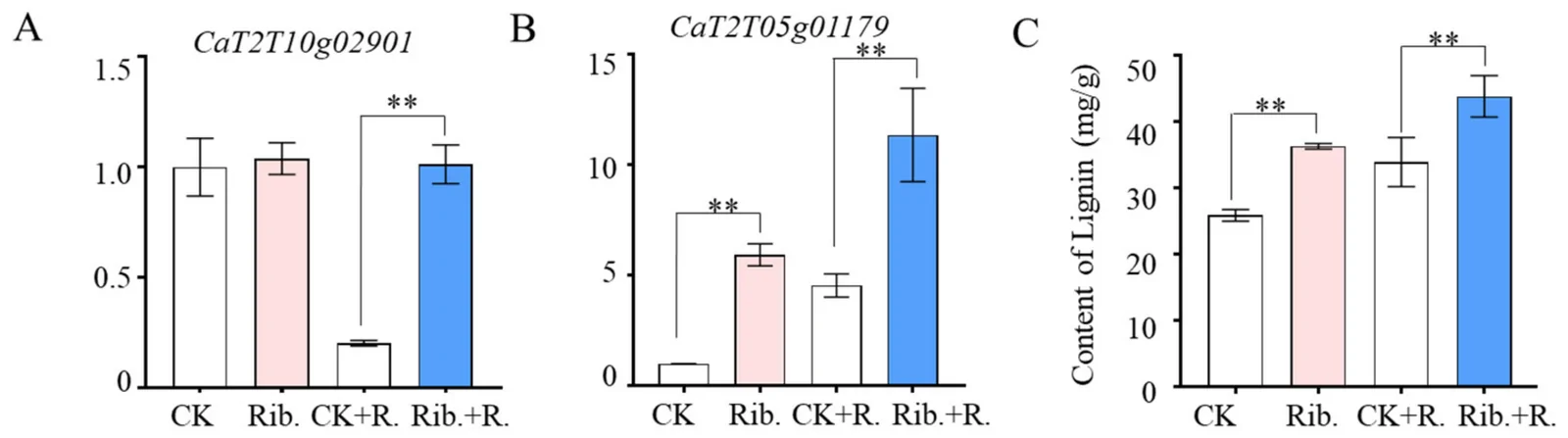
Exogenous riboflavin significantly enhanced accumulation of lignin and gene expression in *NPR1* and *CALM*. (**A**) Upregulated genes related to *NPR1* (*CaT2T10g02901*) under riboflavin treatment. (**B**) Upregulated genes related to *CALM* (*CaT2T05g01179*) under riboflavin treatment. (**C**) Increased accumulation of lignin under riboflavin treatment. Rib represents riboflavin; R represents *R. solanacearum*. CK represents control plants pretreated with water. ** *p* < 0.01.

Among the defense-associated pathways, the expression of *NPR1* gene related to the jasmonic acid signal transduction and *CALM* gene related to plant–pathogen interaction were significantly upregulated, which concurs with recent studies showing that *NPR1* and *CALM* play important roles in resistance to *R. solanacearum* in plants [36,37]. Under unchallenged conditions, riboflavin had little effect on the transcript abundance of *NPR1* (*CaT2T10g02901*) which is involved in plant hormone signaling transduction. Challenge with *R. solanacearum* markedly suppressed *NPR1* expression in non-pretreated plants, whereas riboflavin pretreatment restored its expression to the basal level (Figure 5A). Thus, riboflavin did not constitutively activate *NPR1* but prevented its pathogen-induced suppression, consistent with a priming effect on *NPR1*-associated defense signaling.

By contrast, *CALM* (*CaT2T05g01179*), which plays an important role in plant–pathogen interaction and induced the nitric oxide synthase (NOS), was strongly induced by riboflavin under both unchallenged and pathogen-challenged conditions (Figure 5B). Its transcript abundance was highest in riboflavin-pretreated plants following *R. solanacearum* challenge and was significantly higher than that in challenged plants without riboflavin pretreatment. These findings suggest that riboflavin enhances calcium-associated defense signaling and increases its responsiveness to pathogen challenge.

Research showed the cutin, suberin, and wax biosynthesis pathway was correlated with lignin accumulation [38]. Therefore, lignin content was assessed in this study. Results showed riboflavin also significantly increased lignin content under both conditions (Figure 5C), from approximately 27 to 38 mg g^−1^ in unchallenged plants and from approximately 36 to 46 mg g^−1^ following *R. solanacearum* challenge. Although pathogen challenge alone also increased lignin accumulation, riboflavin pretreatment produced a further significant increase. Collectively, these results indicate that riboflavin enhances defense competence by maintaining *NPR1* and *CALM* expression during pathogen challenge, and reinforcing lignin-based structural barriers.

## 3. Discussion

Riboflavin is an essential precursor of flavin mononucleotide and flavin adenine dinucleotide, two cofactors involved in numerous redox and metabolic reactions. In addition to its fundamental metabolic functions, increasing evidence indicates that exogenously applied riboflavin can act as an elicitor or priming agent that enhances plant immunity [13,34,39,40,41,42]. For example, riboflavin treatment enhances resistance to bacterial leaf blight, as indicated by changes in chlorophyll fluorescence parameters [43], and can restore disease-suppressive functions of the plant microbiome in the tomato *rin* mutant [44]. In the present study, comparative transcriptomic analysis revealed that riboflavin biosynthesis was impaired in the bacterial wilt-susceptible pepper cultivar HS03 relative to the resistant cultivar HR01. Exogenous riboflavin application partially compensated for this apparent metabolic deficiency and conferred 68.0% protection against *R. solanacearum*. Although the transcriptomic comparison alone does not establish a causal relationship between endogenous riboflavin biosynthesis and cultivar resistance, the convergence of genotype-dependent metabolic differences and the protective effect of exogenous riboflavin strongly supports the involvement of riboflavin metabolism in pepper immunity.

A prominent early response to riboflavin treatment was a transient ROS burst (Figure 2B). ROS have a dual function during plant–pathogen interactions, and can directly reduce bacterial viability by damaging membranes and nucleic acids [45,46]. An early ROS burst has been positively associated with resistance to bacterial wilt in eggplant [47]. However, *R. solanacearum* invades through roots, colonizes xylem vessels, and ultimately causes extensive vascular dysfunction and host tissue damage. Consequently, excessive or persistent ROS accumulation may intensify cellular injury and compromise the ability of the host to contain infection [48,49]. Previous studies have shown that riboflavin can promote superoxide production, hypersensitive response-like cell death, and resistance to tobacco mosaic virus [50]. Similarly, the suppression of INF1-induced radical production and cell death in *NbRibA*-silenced *Nicotiana benthamiana* leaves can be reversed by exogenous riboflavin, indicating that flavin metabolism contributes to the regulation of immune-associated ROS production [51]. Our results extend these observations to the pepper–*R. solanacearum* interaction and suggest that riboflavin initiates a controlled oxidative signal rather than inducing sustained oxidative stress.

The early ROS burst was accompanied by rapid changes in antioxidant enzyme activities. SOD, CAT, and POD activities increased at 6 h after riboflavin application, while riboflavin-pretreated plants exhibited greater increases in POD, APX, and CAT activities at 6 h following *R. solanacearum* inoculation (Figure 2 and Figure 3). These enzymes perform complementary functions in ROS homeostasis. SOD converts superoxide radicals into H_2_O_2_, whereas CAT and APX remove excess H_2_O_2_ through distinct catalytic systems. Peroxidases have more context-dependent functions because they can participate in both H_2_O_2_ turnover and H_2_O_2_-dependent oxidation of phenolic substrates. The relatively modest response of SOD compared with those of CAT, APX, and POD suggests that the regulation of downstream H_2_O_2_ metabolism, rather than superoxide dismutation alone, may be particularly important in riboflavin-induced resistance. The close temporal association between CAT and APX activities and ROS abundance is consistent with their established roles in preventing uncontrolled oxidative damage [52,53,54,55]. A similar decline in CAT activity during the later stages of *R. solanacearum* infection has been reported in eggplant [56]. In the present study, the reductions in antioxidant enzyme activities, such as POD and APX, were observed at 48 hpi (Figure 3). This trend was consistent with lower ROS accumulation at 6 hpi in riboflavin-pretreated plants (Figure 1). This pattern may therefore indicate a reduced requirement for ROS detoxification or a return toward redox homeostasis after the early defense response. In our study, riboflavin pretreatment induced ROS burst (Figure 1), and what is more important, it may establish a primed cellular state in pepper leaves. Upon *R. solanacearum* inoculation, this primed state rapidly activated the pre-upregulated enzymatic antioxidant system that was preconditioned by the applied riboflavin, significantly enhancing the activities of SOD, CAT and POD within a short period (Figure 3). These highly accumulated antioxidant enzymes efficiently scavenged the excess hydrogen peroxide generated by both pathogenic infection and riboflavin pretreatment, resulting in a relatively low detectable ROS level. In contrast, the water-infiltrated control samples, which received the same riboflavin pretreatment but no subsequent pathogen challenge, did not trigger this activation of the antioxidant system. As a result, a relatively higher level of H_2_O_2_ accumulation was observed in the samples treated without *R. solanacearum* (Figure 1). Nevertheless, enzyme activity data provide only a snapshot of the overall redox network. Measurements of individual ROS, antioxidant metabolites, and the subcellular localization of ROS will be required to determine precisely how riboflavin regulates ROS production and scavenging.

In addition to regulating ROS homeostasis, riboflavin promotes transcriptional and metabolic responses associated with cell-wall reinforcement. Transcriptome analysis at 12 hpi revealed the differential regulation of genes involved in cutin, suberin, and wax biosynthesis, galactose metabolism, and tyrosine metabolism. These pathways collectively indicate extensive reprogramming of extracellular barrier formation, cell-wall metabolism, and specialized metabolism. Previous studies have demonstrated the cutin, suberin, and wax biosynthesis pathway shared common regulatory target genes with the lignin accumulation pathway, which is directly modulated by the transcription factor QsMY1B in *Quercus suber* [38]. This functional association has also been validated in pear and sugarcane [57,58]. However, pathway enrichment reflects coordinated transcriptional changes, while it cannot demonstrate increased metabolic flux. The observed increasement in lignin content provides complementary biochemical evidence that cell-wall reinforcement was indeed activated in riboflavin-pretreated plants (Figure 4).

Lignin deposition is an important component of plant defense because it strengthens secondary cell walls, restricts pathogen movement, and reduces the accessibility of wall polysaccharides to pathogen-derived enzymes [59,60]. Riboflavin has previously been reported to induce secondary metabolite accumulation [61,62,63], and to prime the phenylpropanoid pathway during rice resistance to *Rhizoctonia solani* [31]. In our study, riboflavin also altered the expression of genes associated with immune signaling. The expression of *NPR1*, a central regulator of salicylic acid-associated defense, was relatively stable following riboflavin treatment alone but decreased following *R. solanacearum* inoculation. Notably, riboflavin pretreatment prevented this pathogen-associated reduction and restored *NPR1* expression during infection. This response is more consistent with defense priming than with constitutive activation, because riboflavin maintained the capacity for a stronger response after pathogen challenge without continuously activating *NPR1* expression in the absence of infection. The *CALM* gene showed a more direct response to riboflavin, and its highest expression occurred in riboflavin-pretreated plants following pathogen inoculation. Because calmodulins translate transient cytosolic Ca^2+^ signals into downstream immune responses, enhanced *CALM* expression may increase the capacity of pepper cells to perceive and transmit pathogen-induced calcium signals. Together, these expression patterns suggest that riboflavin-mediated resistance involves coordinated ROS, calcium, and hormone-associated signaling. However, measurements of calcium fluxes, salicylic acid accumulation, NPR1 protein abundance, and downstream defense-gene expression will be necessary to confirm this regulatory connection.

Collectively, our findings support a biphasic model of riboflavin-induced resistance in pepper. Riboflavin first elicits transient ROS accumulation and enhances the responsiveness of calcium- and defense-associated signaling pathways. It subsequently activates antioxidant enzymes that constrain excessive ROS accumulation while promoting transcriptional reprogramming and lignin deposition. This coordinated regulation enables ROS to function as a defense signal without causing prolonged oxidative injury and ultimately strengthens pepper resistance to *R. solanacearum*. Riboflavin therefore represents a promising environmentally compatible priming agent for bacterial wilt management. Future studies should distinguish its host-mediated effects from any direct antibacterial activity and evaluate dose dependence, the duration of protection, field efficacy, and the functional contributions of specific peroxidase isoforms and riboflavin-biosynthetic genes.

## 4. Materials and Methods

### 4.1. Plant and Pathogen Material

Two representative pepper inbred lines, HR01 (resistant) and HS03 (susceptible), propagated by our lab were selected for this study. The pepper seedlings were cultivated at 28 °C under a light cycle of 16 h of light and 8 h of darkness. Three biological replicates were performed, each replicate including at least three seedlings.

*R. solanacearum* strain R3-1 was originally isolated from diseased pepper plants in Fuzhou, Fujian, China; its genomic sequence was compared with *R. solanacearum* strain MT758110.1 (GenBank accession: MT758110.1) and other bacterial species (Figure S5). The 16S rRNA regions were sequenced using the primer pair listed in Table S1 to confirm identity, and sequencing was performed by Beijing Meiyouanno Biotechnology Co., Ltd. (Beijing, China).

The leaf injection method was used for inoculation [64]. Briefly, the seedlings at the fifth-leaf–one-heart stage were selected, and the third healthy true leaves of uniform size and node position were chosen for injection. *R. solanacearum* was prepared at OD_600_ = 0.3 in 10 mM MgCl_2_ solution (pH 5.6). Control plants were infiltrated with sterile 10 mM MgCl_2_ solution (pH 5.6). A small wound was made on the abaxial epidermis by gentle friction before injection. Each treatment group included three biological replicates, with at least six seedlings per replicate. Seedlings were grown in a greenhouse at 30 °C, 75% relative humidity, under a 16 h light/8 h dark cycle after inoculation.

### 4.2. Riboflavin and Inoculation Treatment Methods

The HS03 plants were divided into 3 groups, pretreated with different concentrations (0, 0.2, 1 mM) of riboflavin. The leaves of the plants in each subgroup were sprayed 2 times (about 50 mL solution for each group) during the first 4 days (the 1st and 4th day). Then each group was further divided into 2 subgroups: water (as control), and *R. solanacearum* group (at least 12 plants for each subgroup). The third, fourth and fifth leaves of single pepper seedlings of each group were harvested from 0, 6 and 48 h for enzyme activity assay, and the fifth leaves were chosen for analysis for ROS content. Fresh leaves were then stored at −70 °C for subsequent analyses. The leaf injection inoculation method was used as described above. Seedlings at the sixth-leaf–one-heart stage were selected for the bacterial injection assay, and the third and fourth healthy true leaves, with similar leaf size and consistent node placement, were selected for inoculation 6 h after riboflavin application. Concentration of *R. solanacearum* was prepared at OD_600_ = 0.3, and a 10 mM MgCl_2_ solution (pH = 5.6) was used as inoculation solution. Control treatments involved identical procedures, with seedlings infiltrated using sterile 10 mM MgCl_2_ solution (pH = 5.6). The underside of the leaf was subjected to pressure infiltration with a needleless syringe and approximately 0.05 mL of the bacterial suspension was injected to create an infection. A small wound was created on the abaxial epidermis by gentle friction before injection. Each treatment group included four biological replicates, and twelve seedlings were used per genotype. Inoculated seedlings were cultivated normally in a greenhouse at 30 ± 5 °C with 75% relative humidity, under a light cycle of 16 h of light and 8 h of dark.

### 4.3. Disease Assessment and Pathogen Identification

Disease symptoms were monitored at 2, 3, 5, 7, and 10 days post-inoculation (dpi). Based on the progression of wilt symptoms, disease severity tended to stabilize around 7 dpi. Therefore, resistance evaluation (survival seedlings/total seedlings) was performed at this time point. The disease symptoms were divided into two scales based on their disease severity: no wilt symptoms and significant wilt symptoms.

### 4.4. RNA Sample Collection, RNA-Seq Library Construction, Sequencing and Analysis

Seedlings from inbred lines HR01 (resistant) and HS03 (susceptible) were prepared and inoculated at the six-leaf stage in an incubator (PGX-350L, Ningbo Saifu Experimental Instrument Co., Ltd., Ningbo, China) at 30 ± 5 °C under a 16/8 h light/dark photoperiod and 75% humidity, and sampled at 0 and 6 h after inoculation for transcriptomic analyses. Three grams of each sample were collected from the third leaf and instantly frozen in liquid nitrogen and stored at −80 °C until RNA extraction. Three biological replicates were performed for RNA extraction using poly-T oligo-attached magnetic beads, and a NEBNext^®^ Ultra™ RNA Library Prep Kit for Illumina^®^ was used to construct libraries (NEB, Ipswich, MA, USA). Sequencing was performed with an Illumina HiSeq 4000 platform to obtain the 150 bp paired-end reads. Differentially expressed genes (DEGs) were identified from the differential expression analysis using DESeq2 R package (version 1.52.0) and the hierarchical cluster analysis. The significant DEGs were filtered based on FDR (false discovery rate) ≤ 0.01 and |log_2_ fold change| ≥ 1 in each pairwise comparison, and adjusted *p* value ≤ 0.05 identified DEGs. Functional enrichment analysis of the DEGs was performed using the Gene Ontology (GO) and Kyoto Encyclopedia of Genes and Genomes (KEGG) databases. Pathways with an adjusted *p* value ≤ 0.05 were considered significantly enriched and a heat map of expression profiles was constructed using TB-tools (version 1.108). Three biological replicates and four technical replicates were performed for each qRT-PCR analysis, and 2^−ΔΔCT^ was used to calculate gene expression. The gene-specific primers for qRT-PCR are listed in Table S1, annotated and designed based on the pepper genome database (bioinformaticslab.cn) [65].

### 4.5. Measurement of H_2_O_2_ Production

Total H_2_O_2_ in the leaf was measured according to Bellincampi et al. [66]. Briefly, 2 g of tissue was weighed and immediately quenched in liquid nitrogen. Samples were homogenized in 50 mM sodium phosphate buffer (pH 7.0), and 1 mL of the supernatant was collected by centrifugation at 10,000× *g* for 20 min at 25 °C; an aliquot of supernatant (500 µL) was added to 500 mL of assay reagent (500 mM ferrous ammonium sulfate, 50 mM H_2_SO_4_, 200 mM xylenol orange, and 200 mM sorbitol). After 45 min of incubation, the peroxide-mediated oxidation of Fe^2+^ to Fe^3+^ was determined by measuring the A_560_ of the Fe^3+^ xylenol orange complex.

### 4.6. Antioxidant Enzyme Assay

A 0.5 g fresh leaf tissue sample was ground to powder in liquid nitrogen and homogenized in 3 mL ice-cold extraction buffer which consisted of 0.1 M Tris-HCl (pH 7.5), 0.23 M sucrose, 5% polyvinylpyrrolidone (PVP), 1 mM EDTA, 10 mM KCl, 10 mM MgCl_2_, and 2.5 mM ascorbic acid (added to the buffer just before use). After homogenization and centrifugation (10,000× *g* for 30 min), the supernatants were used for enzyme assays. SOD activity was determined by NBT as described by Janknegt et al. [67]. For SOD activity detection, the reaction buffer, containing 13 mM methionine, 75 μM NBT, 100 μM ethylenediaminetetraacetic acid (EDTA) and 2 μM riboflavin, was mixed with different volumes of enzyme extract in 50 mM phosphate buffer (pH 7.8). Then the mixture was exposed to light illumination for 15 min. The increase in absorbance due to formazan formation was read at 560 nm. One unit of SOD activity is defined as the amount of enzyme that inhibits the NBT photoreduction by 50%. The CAT, APX and POD activities were measured using the method described by El-Marakby et al. [68]. For CAT activity detection, each 3 mL reaction mixture contained 150 mM potassium phosphate buffer (pH 7.0), 15 mM H_2_O_2_, and 50 μL enzyme extract. The reaction was initiated by adding 15 mM H_2_O_2_. The activity was determined by monitoring the consumption of H_2_O_2_ (E = 39.4 mM^−1^ cm^−1^) at 240 nm for 3 min. For APX activity detection, the reaction mixture (1 mL) was composed of 50 mM K-phosphate buffer (pH 7.0), 0.1 mM H_2_O_2_, 0.5 mM Na-ascorbate, and 0.1 mM EDTA. The hydrogen peroxide-dependent oxidation of ascorbate was measured by a decrease in the absorbance at 290 nm (ε = 2.8 mM^−1^ cm^−1^). Activity of APX was expressed as U/g under the assay conditions as described [33]. Peroxidase (POD) activities were determined by measuring the absorbance changes at 470 nm and 25 °C with some modification. The reaction was performed in a 3 mL solution: a 10 µL volume of enzyme solution was added to 2.99 mL of sodium phosphate buffer (50 mM, pH 6.0) containing 18.2 mM guaiacol and 4.4 mM H_2_O_2_ as substrates [69].

### 4.7. Lignin Determination

Lignin was extracted and measured as previously described [70]. Briefly, samples were collected at 48 hpi and dried at 105 °C, then lignin content was calculated from absorbance at 280 nm. Three biological replicates and three technical replicates were performed for all biochemical assays.

### 4.8. Data Analysis

All data were obtained from three independent biological replicates and are presented as the mean ± standard error (mean ± SE). Statistical significance was assessed using Student’s *t*-test (*p* < 0.05) or Tukey’s multiple comparisons test with SPSS version 25.0 software (IBM Corp., Armonk, NY, USA).

## Data Availability

The original contributions presented in this study are included in the article/Supplementary Material. Further inquiries can be directed to the corresponding authors.

## Supplementary Materials

The following supporting information can be downloaded at: https://www.mdpi.com/article/10.3390/ijms27209007/s1.
