# Supplementary material for: Riboflavin Primes Pepper Resistance to Ralstonia solanacearum Through Coordinated Antioxidant Responses and Lignin Deposition
Source: Int J Mol Sci. 2026 Oct 11;27(20):9007. doi: 10.3390/ijms27209007 (PMC13704953; doi:10.3390/ijms27209007)
Supplement: Supplementary file 1 [file ijms-27-09007-s001.zip › Supplementary Materials (Table S1; Figure S1-S5).pdf]

Table S1. *Primers used* in *this study.*

**Gene ID**	**Primer-F**	**Primer-R**	**annotation**
R3-1	GTCGCCGTCAACTCACTTTCC	GTCGCCGTCAGCAATGCGGAATCG	Strain identification
CaT2T05g00598	TCCTGAGGAGCTTGCCATTG	GTGTCTGTGGCTACTGCTGT	*CaATPF1A*
CaT2T07g00935	ACAGTGGCTTTCAGCTCCTC	CCAAAGCGGCGAAGAACAAA	*CaND2*
CaT2T11g01196	TCGATTCTGATAGGTGCGCC	TGATACCGGATCCAGGCAGA	*CaCOX3*
CaT2T10g02901	AGCGCTCGACTCATCTGATG	TCCCACGTTCGTGTTGGTAG	*CaNPR1*
CaT2T05g01179	ACGAAAATCCAACGCCCGTA	CCTCTCCGAGGCTCTGGATA	*CaCALM*
CaT2T08g02441	ACCTACTATGTGGGGCCCAT	TGTTGGTTGTCGGGCGAATA	*CaDHPB*
CaT2T06g03488	TGCAAAAGGATCCTCCCACA	CTGAGTCCAGCTACGAGCAG	CaUBI-quintin, Reference genes
27F	AGAGTTTGATCMTGGCTCAG	TACGGYTACCTTGTTACGACTT	For R. s identification

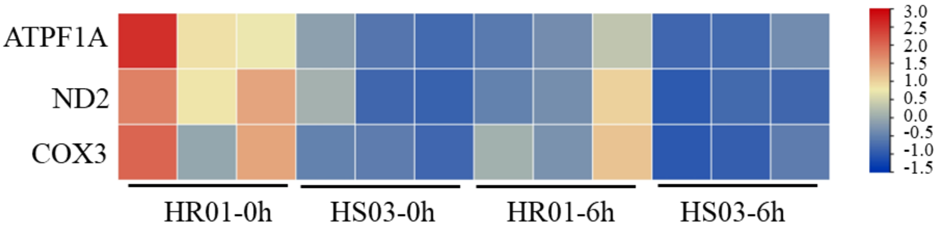

Figure S1. Heatmap of genes in oxidative phosphorylation pathway in HR01 (resistant cultivar) compared to HS03 (susceptible cultivar) 0 hpi or 6hpi

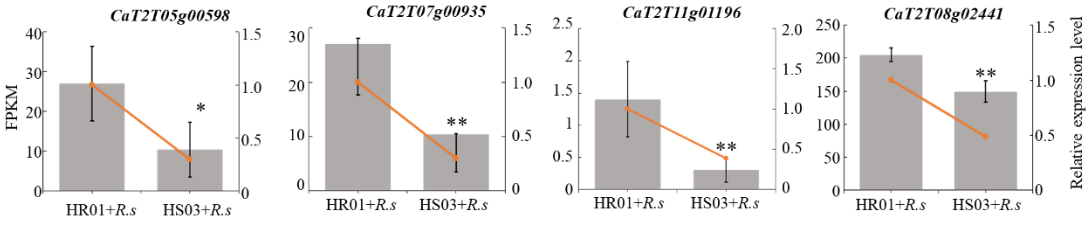

Figure S2. Expression of genes under control or Riboflavin treatment as measured by qRT-PCR (orange symbols) and RNA-seq (gray bars) in oxidative phosphorylation pathway and riboflavin synthesis pathway in HR01 (resistant cultivar) compared to HS03 (susceptible cultivar) 6hpi. For qRT-PCR, gene expression levels were normalized to those of *CaUBI,* qRT-PCR expression levels in the variety treated with control (HR01+*R.s*) were set to 1. Data are presented as the means ± SE of three biological replicates. **p*<0.05, * *p* < 0.01 (Student's *t*-test, qRT-PCR data)

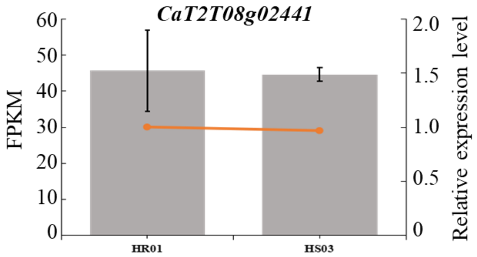

Figure S3. Expression of genes under control as measured by qRT-PCR (orange symbols) and RNA-seq (gray bars) in riboflavin synthesis pathway in HR01 (resistant cultivar) compared to HS03 (susceptible cultivar) at 0 hpi (hours post inoculation). For qRT-PCR, gene expression levels were normalized to those of *CaUBI,* qRT-PCR expression levels in the variety treated with control (HR01) were set to 1. Data are presented as the means ± SE of three biological replicates. **p*<0.05, * *p* < 0.01 (Student's *t*-test, qRT-PCR data)

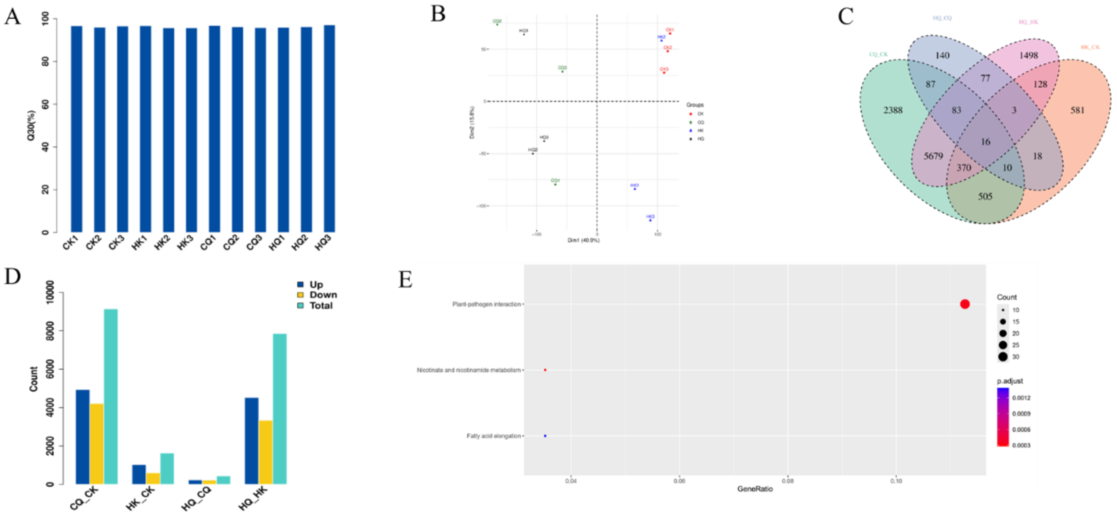

Figure S4. Overview of RNA-seq quality control and gene expression profile characteristics. (A) Q30 base quality distribution of all RNA-seq samples; (B) Principal component analysis (PCA) plot of gene expression profiles across all samples; (C) Venn diagram showing the overlap and specificity of expressed genes among different groups, (n=3, three biological replicates per group); (D) Statistics of differentially expressed genes (DEGs) across four pairwise comparison groups; (E) KEGG analysis of HS03 under riboflavin treatment compared with control.

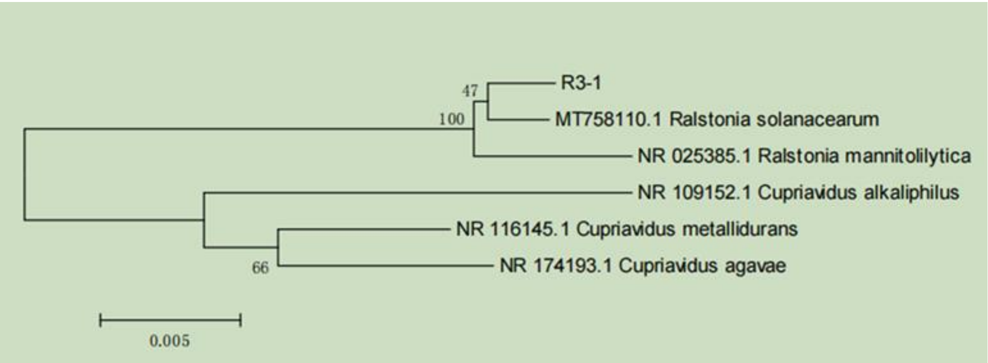

Figure S5 Strain identification result showed R3-1 is *R. solanacearum.*
